# Longitudinal relationship of favorable weight change to academic performance in children

**DOI:** 10.1038/s41539-020-0063-z

**Published:** 2020-04-24

**Authors:** Toru Ishihara, Toshihiro Nakajima, Koji Yamatsu, Koichi Okita, Masato Sagawa, Noriteru Morita

**Affiliations:** 1 Tamagawa University Brain Science Institute, Tokyo, Japan; 2 Japan Society for the Promotion of Science, Tokyo, Japan; 3 Department of Teachers Training, Hokkaido University of Education, Hokkaido, Japan; 4 Faculty of Education, Saga University, Saga, Japan; 5 Department of Sport Education, Hokusho University, Hokkaido, Japan; 6 Department of Sports Cultural Studies, Hokkaido University of Education, Hokkaido, Japan

**Keywords:** Human behaviour, Learning and memory

## Abstract

Although there is a growing consensus about the positive relationship between prevention of overweight/obesity and academic performance in children, relevant studies targeting the relationship between underweight and academic performance are scarce. This study aimed to examine the longitudinal relationship of favorable weight change to academic performance in schoolchildren. We analyzed 2-year longitudinal data derived from 197 seventh-grade children aged 12–13 years. Academic performance was assessed using the total grade points of five academic subjects. Body mass index (BMI) was calculated as body weight (kg)/height (m^2^). A significant interaction effect of baseline BMI and BMI changes over 2 years (*B* = −0.10, SE *B* = 0.03, *β* = −0.40, *t* = –3.37, *p* < 0.001) was noted after controlling for confounders such as socioeconomic status, afterschool learning duration, screen time, exercise habits, and cardiorespiratory fitness. When the centered baseline BMI was outside the interval [−2.49, 3.21], the slope of the change in BMI was significant (*p* < 0.05). Simple slope analyses revealed a positive relationship of weight gain when baseline BMI = mean − 1 SD (*B* = 0.40, SE *B* = 0.18, *β* = 0.31, *t* = 2.20, *p* = 0.03) and weight loss when baseline BMI = mean + 1 SD (*B* = −0.26, SE *B* = 0.13, *β* = −0.20, *t* = −1.97, *p* = 0.05) to total grade points of five school subjects. A split-group validation was performed and robust results of original analyses were detected (i.e., significant interaction effect of baseline BMI and BMI changes over 2 years (group A: *B* = −0.11, SE *B* = 0.05, *β* = −0.47, *t* = −2.39, *p* = 0.02; group B: *B* = −0.14, SE *B* = 0.05, *β* = −0.47, *t* = −2.78, *p* = 0.007). Favorable changes in weight status, i.e., weight loss in children with overweight/obesity and weight gain in children with mild underweight/underweight, have a positive influence on academic performance in children independent of socioeconomic factors, learning habits, screen time, exercise habits, and cardiorespiratory fitness.

## Introduction

The incidence of childhood overweight and obesity over the past three decades and the associated health problems are increasing worldwide, which in turn has become a public health concern among educators and policymakers^1–4^. Childhood overweight and obesity are associated with risk of diseases such as cardiovascular diseases and type 2 diabetes^1,4^, lower academic performance, and poor brain health^5–9^. For example, higher body mass index (BMI) and fat mass are associated with lower academic performance and decreased ability to perform higher-order cognition among children^7–9^. Previous studies reported that higher-order cognition, called “executive function,” referring to a family of top-down mental processes needed for goal-directed behavior, was closely associated with learning and academic performance^10–12^. Kamijo et al.^7–9^ demonstrated that obese children simultaneously showed poor academic performance and poor executive function task performance. For example, obese children showed lower ability to modulate the cognitive control network, to optimize behavioral interactions within the environment^8^. Thus, there appears to be empirical evidence that a healthy lifestyle contributing to maintaining a healthy weight status improves executive function and subsequently academic performance of children.

Childhood underweight is well known to cause other health problems^13^. For example, childhood underweight has been associated with decreased immune function and increased risk of infectious disease^13^. However, to our knowledge, studies that examine the association of childhood underweight to academic performance and cognitive health are limited. Given that the prevalence of childhood underweight is more than 20% in some Asian countries^1^, thereby making it a serious public health concern in Asian countries^1–3^, how underweight status affects academic performance in Asian children needs to be clarified.

Underweight children are presumed to have disadvantages such as malnutrition and slow growth. Given that children with malnutrition show poor executive function^14,15^, underweight children could also show poor academic performance. Kar et al.^14^ showed that children with malnutrition demonstrated poor cognitive task performance requiring executive function. In an animal study, malnutrition intervention reduced brain weight associated with poor executive function in mice^16^. Although the mechanisms may be different, both overweight/obesity and underweight could impair executive function and, in turn, cause poor academic performance.

Indeed, a previous study suggests the possibility of a negative relationship between childhood underweight and academic performance^17^. More specifically, the study showed an inverted U-shape relationship between BMI and academic performance, suggesting that both childhood underweight and overweight are associated with lower academic performance^17^. However, the study employed a cross-sectional design and could not illustrate causal inference. Thus, a longitudinal study investigating the effects of favorable change in weight status, i.e., weight gain for children with underweight and weight loss for children with overweight/obesity, on academic performance in children is warranted.

To this end, this 2-year longitudinal study aimed to investigate the relationship of weight status to academic performance of Japanese children after controlling for confounders (i.e., socioeconomic status, cardiorespiratory fitness, exercise habits, screen time, and learning duration). Moreover, this study focused on the two directions of weight change, i.e., weight gain in children with underweight and weight loss in children with overweight, to provide new insights into how a healthy lifestyle could influence the academic performance.

## Results

Participants’ demographic data are presented in Table 1.

**Table 1:** Characteristics of the study participants.

	Baseline	2-Year follow-up	Effect size
*N*	197		
Boys (*N* (%))	98 (50)		
Girls (*N* (%))	99 (50)		
Socioeconomic status			
Component score	3 ± 1 (2 to 5)		
Household income (*N* (%))			
Less than 2,000,000 yen	7 (4)		
2,000,000 yen to 4,000,000 yen	26 (13)		
4,000,000 yen to 6,000,000 yen	64 (32)		
6,000,000 yen to 8,000,000 yen	72 (37)		
More than 8,000,000 yen	28 (14)		
Maternal education (*N* (%))			
Junior high school	2 (1)		
High school	99 (50)		
Vocational school	48 (24)		
Junior college	29 (15)		
Undergraduate studies	19 (10)		
Graduate studies	0 (0)		
Total grade points of academic subjects	18 ± 4 (8 to 25)	18 ± 4 (6 to 25)	0.01
Japanese	4 ± 1 (1 to 5)	4 ± 1 (2 to 5)	
Mathematics	4 ± 1 (1 to 5)	4 ± 1 (1 to 5)	
Social studies	4 ± 1 (1 to 5)	4 ± 1 (1 to 5)	
Science	4 ± 1 (1 to 5)	4 ± 1 (1 to 5)	
English	4 ± 1 (2 to 5)	4 ± 1 (1 to 5)	
BMI (kg/m^2^)	19 ± 3 (13 to 30)	20 ± 3 (14 to 32)	0.30***
Cardiorespiratory fitness	5 ± 2 (1 to 10)	7 ± 2 (2 to 10)	0.92***
Exercise habits			
Component score	5 ± 2 (1 to 7)	4 ± 2 (1 to 7)	−0.22***
Frequency (*N* (%))			
No	23 (12)	38 (19)	
1 Day/week	14 (7)	20 (10)	
2 Days/week	15 (8)	14 (7)	
3 Days/week	7 (4)	8 (4)	
4 Days/week	7 (4)	8 (4)	
5 Days/week	22 (11)	19 (10)	
6 Days/week	32 (16)	34 (17)	
7 Days/week	77 (39)	56 (28)	
Duration on weekdays (*N* (%))			
Less than 15 min	37 (19)	55 (28)	
15 to 30 min	7 (4)	17 (9)	
30 min to 1 h	19 (10)	13 (7)	
1 to 2 h	51 (26)	22 (11)	
2 to 3 h	64 (32)	57 (29)	
3 to 4 h	12 (6)	22 (11)	
Over 4 h	7 (4)	11 (6)	
Duration on weekends (*N* (%))			
Less than 15 min	31 (16)	48 (24)	
15 to 30 min	12 (6)	13 (7)	
30 min to 1 h	14 (7)	20 (10)	
1 to 2 h	14 (7)	12 (6)	
2 to 3 h	33 (17)	20 (10)	
3 to 4 h	50 (25)	46 (23)	
Over 4 h	43 (22)	38 (19)	
Screen time			
Component score	2 ± 1 (1 to 5)	2 ± 1 (1 to 5)	−0.26***
TV (*N* (%))			
Over 2 h	79 (40)	75 (38)	
1 to 2 h	70 (36)	84 (43)	
30 min to 1 h	28 (14)	20 (10)	
15 to 30 min	11 (6)	12 (6)	
Almost no watching	9 (5)	6 (3)	
Electronic devices (*N* (%))			
Over 2 h	39 (20)	66 (34)	
1 to 2 h	45 (23)	55 (28)	
30 min to 1 h	39 (20)	23 (12)	
15 to 30 min	32 (16)	19 (10)	
Almost no using	42 (21)	34 (17)	
Learning duration			
Component score	3 ± 1 (1 to 5)	4 ± 1 (1 to 5)	0.64***
Duration on weekdays (*N* (%))			
Less than 30 min	20 (10)	12 (6)	
30 min to 1 h	32 (16)	17 (9)	
1 to 2 h	78 (40)	44 (22)	
2 to 3 h	46 (23)	62 (31)	
Over 3 h	21 (11)	62 (31)	
Duration on weekends (*N* (%))			
Less than 30 min	27 (14)	18 (9)	
30 min to 1 h	26 (13)	11 (6)	
1 to 2 h	66 (34)	36 (18)	
2 to 3 h	51 (26)	37 (19)	
Over 3 h	27 (14)	95 (48)	

*BMI* body mass index.

Values are presented as *N* (%) or mean ± SD (range); paired *t*-test was performed to compare baseline and 2-year follow-up values; ***p < 0.001.

### Main analysis

**Fig. 1:**
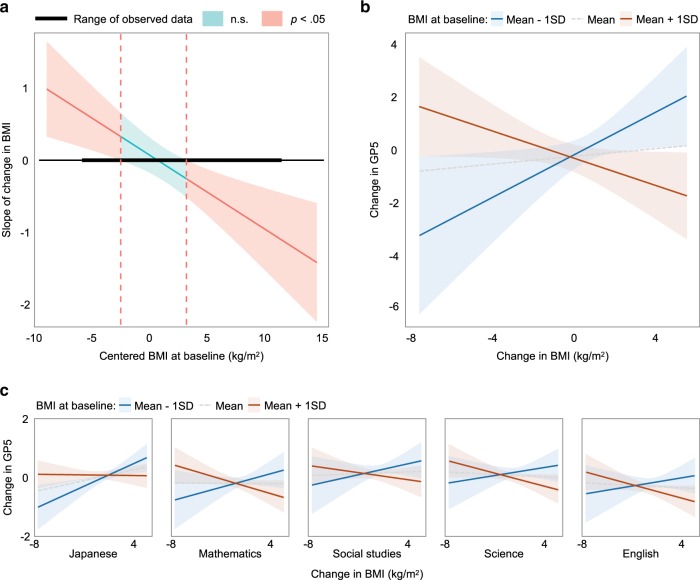
Relationship between changes in BMI and academic performance. **a** Johnson–Neyman Interval. A significant interaction effect of BMI at baseline and change in BMI over 2 years was detected (*B* = −0.10, SE *B* = 0.03, *β* = −0.40, *t* = −3.37, *p* < 0.001). When centered baseline BMI was outside the interval [−2.49, 3.21], the slope of change in BMI was significant. **b** Results of simple slope analysis. The slope analyses revealed a positive relationship of weight gain when baseline BMI = mean − 1 SD (*B* = 0.40, SE *B* = 0.18, *β* = 0.31, *t* = 2.20, *p* = 0.03) and weight loss when baseline BMI = mean + 1 SD (*B* = −0.26, SE *B* = 0.13, *β* = −0.20, *t* = −1.97, *p* = 0.05) to the total grade points of the five school subjects. **c** The results of simple slope analysis for each academic subject. The pattern of the interaction effect of BMI at baseline and change in BMI over 2 years on academic performance was robustly found across academic subjects.

The results of the hierarchical regression analysis are presented in Table 2. Steps 1 and 2 of the regression analysis predicting the total grade points of the five school subjects were not significant (Δ*R*^2^ < 0.02, *p* > 0.39). Step 3 revealed a significantly increased coefficient of determination (Δ*R*^2^ = 0.15, *p* < 0.001). Significant effects of changes in screen time (*B* = 0.48, SE *B* = 0.19, *β* = 0.22, *t* = 2.57, *p* = 0.01) and learning duration (*B* = 0.68, SE *B* = 0.18, *β* = 0.34, *t* = 3.82, *p* < 0.001) over 2 years were noted. Step 4 also revealed a significantly increased coefficient of determination (Δ*R*^2^ = 0.06, *p* = 0.02) and a significant interaction effect of BMI at baseline and change in BMI over 2 years (*B* = −0.10, SE *B* = 0.03, *β* = −0.40, *t* = −3.37, *p* < 0.001) was detected. The Johnson–Neyman Interval is shown in Fig. 1a. When centered baseline BMI was outside the interval [−2.49, 3.21], the slope of change in BMI was significant (Fig. 1a; *p* < 0.05). Simple slope analyses revealed a positive relationship of weight gain when baseline BMI = mean − 1 SD (*B* = 0.40, SE *B* = 0.18, *β* = 0.31, *t* = 2.20, *p* = 0.03) and weight loss when baseline BMI = mean + 1 SD (*B* = −0.26, SE *B* = 0.13, *β* = −0.20, *t* = −1.97, *p* = 0.05) to the total grade points of the five school subjects (Fig. 1b). This pattern (i.e., interaction effect of BMI at baseline and change in BMI over 2 years) was robustly found across academic subjects (Fig. 1c; Japanese: *B* = −0.02, SE *B* = 0.008, *β* = −0.28, *t* = −2.65, *p* = 0.009; Mathematics: *B* = −0.02, SE *B* = 0.01, *β* = −0.27, *t* = −2.40, *p* = 0.02; Social studies: *B* = −0.02, SE *B* = 0.01, *β* = −0.19, *t* = −1.62, *p* = 0.11; Sciences: *B* = −0.02, SE *B* = 0.009, *β* = −0.23, *t* = −1.98, *p* = 0.05; and English: *B* = −0.02, SE *B* = 0.01, *β* = −0.22, *t* = −1.92, *p* = 0.06).

**Table 2:** Hierarchical regression models predicting change in total grade points of five academic subjects.

	Step 1				Step 2				Step 3				Step 4			
	*B*	SE *B*	*β*	*t*	*B*	SE *B*	*β*	*t*	*B*	SE *B*	*β*	*t*	*B*	SE *B*	*β*	*t*
Sex	−0.003	0.31	−0.001	−0.01	−0.07	0.32	−0.02	−0.20	0.03	0.31	0.006	0.08	0.07	0.30	0.02	0.20
Socioeconomic status	0.27	0.16	0.12	1.64	0.25	0.16	0.12	1.49	0.11	0.15	0.05	0.67	0.06	0.15	0.03	0.40
Grade points at baseline	−0.04	0.04	−0.07	−0.93	−0.04	0.04	−0.07	−0.90	−0.11	0.04	−0.20	−2.53**	−0.12	0.04	−0.21	−2.67**
Exercise habits at baseline					0.11	0.16	0.05	0.59	−0.02	0.22	−0.01	−0.11	−0.05	0.22	−0.02	−0.22
Screen time at baseline					0.16	0.15	0.08	1.00	0.34	0.18	0.16	1.83	0.37	0.17	0.17	2.03*
Learning duration at baseline					0.04	0.17	0.02	0.24	0.46	0.20	0.21	2.24*	0.57	0.20	0.26	2.81**
Cardiorespiratory fitness at baseline					−0.001	0.09	−0.001	−0.01	0.12	0.11	0.11	1.04	0.09	0.11	0.09	0.83
BMI at baseline					−0.008	0.07	−0.01	−0.12	−0.03	0.07	−0.05	−0.49	−0.02	0.07	−0.03	−0.31
Square value of BMI at baseline					0.005	0.01	0.05	0.48	0.01	0.009	0.05	0.56	−0.01	0.01	−0.08	−0.76
ΔExercise habits									0.13	0.23	0.04	0.53	0.10	0.22	0.03	0.40
ΔScreen time									0.48	0.19	0.22	2.57*	0.51	0.17	0.24	2.79**
ΔLearning duration									0.68	0.18	0.34	3.82***	0.70	0.18	0.34	3.94***
ΔCardiorespiratory fitness									0.19	0.13	0.14	1.53	0.12	0.13	0.09	0.97
ΔBMI									−0.11	0.11	−0.09	−1.03	0.07	0.11	0.06	0.58
BMI at baseline × ΔExercise habits													0.08	0.06	0.08	1.10
BMI at baseline × ΔScreen time													−0.05	0.05	−0.06	−0.78
BMI at baseline × ΔLearning duration													−0.06	0.04	−0.10	−1.30
BMI at baseline × ΔCardiorespiratory fitness													−0.04	0.03	−0.10	−1.02
BMI at baseline × ΔBMI													−0.10	0.03	−0.40	−3.37***
*R*^2^				0.02				0.03				0.17**				0.23***
Δ*R*^2^				0.02				0.01				0.15				0.06
*F* change in *R*^2^				1.00				0.37				6.70***				2.74*
AIC				565				575				553				549

*AIC* Akaike’s information criterion; *BMI* body mass index.

Multicollinearity was inconsequential (variance inflation factor values ≤ 3.2); *p < 0 .05; **p < 0.01; ***p < 0.001.

To determine whether there might be problems with the regression model, we confirmed these results using diagnostic plots. We found that there were no problems regarding heteroscedasticity, normality, or influential observations with respect to our regression model (Supplementary Fig. 1A–D).

### Moderation analysis by sex

**Fig. 2:**
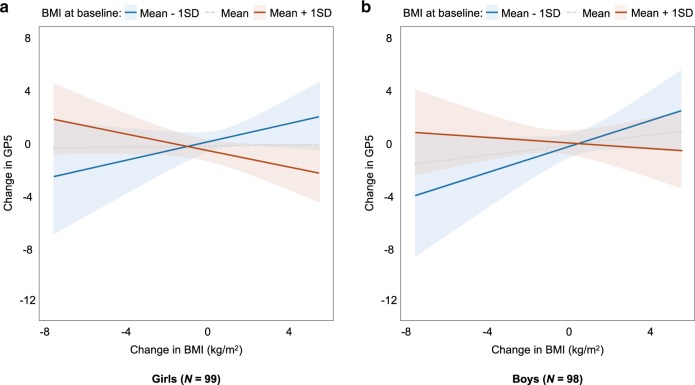
Results of the moderation analysis by sex. Relationship between changes in BMI and academic performance in **a** girls (left panel) and **b** boys (right panel). There was no significant three-way interaction of sex × baseline BMI × change in BMI (*B* = 0.02, SE *B* = 0.06, *β* = 0.03, *t* = 0.37, *p* = 0.71).

To test the moderating effects of sex, we added two- to three-way interaction regarding sex to the regression model. We found no significant effects of sex × BMI × ΔBMI (*B* = 0.02, SE *B* = 0.06, *β* = 0.03, *t* = 0.37, *p* = 0.71). Simple slope analyses showed consistent patterns of main analysis in both boys and girls (Fig. 2).

### A split-group validation analysis

**Fig. 3:**
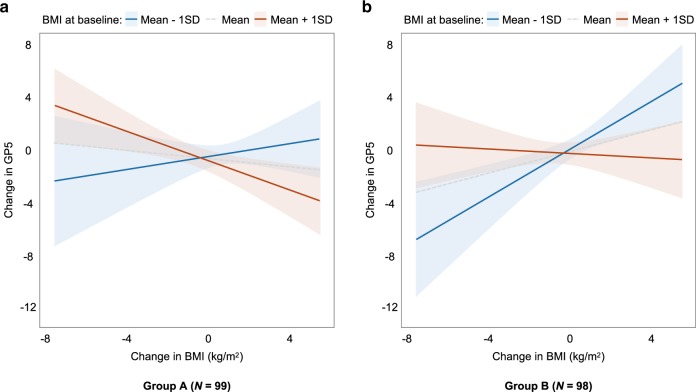
Results of a split-group validation analysis. Relationship between changes in BMI and academic performance in **a** group A (left panel) and **b** group B (right panel). The main findings of a significant interaction effect of baseline BMI and BMI change over 2 years were robustly replicated in both group A (*B* = −0.11, SE *B* = 0.05, *β* = −0.47, *t* = −2.39, *p* = 0.02) and group B (*B* = −0.14, SE *B* = 0.05, *β* = −0.47, *t* = −2.78, *p* = 0.007).

To determine the reproducibility and validity of the current main findings, we separated the participants into two groups and performed hierarchical multiple regression analysis in each group in the same way as the main analyses. We divided the participants using cluster analysis based on baseline BMI and BMI change over 2 years. The participants were divided into three clusters after controlling for sex and socioeconomic status (Supplementary Fig. 2A, B). Then, the subjects were listed according to their baseline BMI values, i.e., from the lowest to highest BMI, in each cluster and divided alternately into two groups (groups A and B; Supplementary Fig. 2C). Demographic data of the participants in each group are presented in Supplementary Table. The main findings of a significant interaction effect of baseline BMI and BMI change over 2 years were robustly replicated in groups A and B (group A: *B* = −0.11, SE *B* = 0.05, *β* = −0.47, *t* = −2.39, *p* = 0.02; group B: *B* = −0.14, SE *B* = 0.05, *β* = −0.47, *t* = −2.78, *p* = 0.007). Simple slope analyses showed consistent patterns of main analysis in both groups A and B (Fig. 3).

## Discussion

Weight loss in Japanese children with overweight/obesity and weight gain in those with underweight were associated with improvement in their academic performance. This positive association of favorable change in weight over 2 years with academic performance was independent of socioeconomic status, exercise habits, screen time, learning duration, and cardiorespiratory fitness. When baseline BMI was outside the interval [mean − 2.49, mean + 3.21], the relationship of change in BMI to academic performance was significantly detected. This result suggests that positive associations of weight optimization are observed not only in underweight and obese children but also in mild underweight and overweight children. In addition, such associations were consistently found regardless of sex and academic subjects. These findings suggest that weight optimization could contribute to broad academic subjects, both in boys and girls. To the best of our knowledge, our study’s results provide the first evidence that weight gain in children with underweight contributes to the improvement in academic performance.

The results of this study indicated that weight loss in children with overweight/obesity is associated with improvement in academic performance, which is consistent with the findings of previous randomized control trials, which showed that physical activity intervention aimed to reduce weight improves brain function and academic performance of children with overweight/obesity^18,19^. For example, in a previous study, a 13-week physical activity intervention (20 or 40 min/day) was associated with improved math achievement among children with overweight aged 7–11 years^18^. Our results are broadly consistent with those of the aforementioned studies revealing that weight loss in children with overweight contributes to academic performance. In addition, given that sleeping habits and dietary intake have been shown to be associated with BMI in children^20–23^, changes in sleeping habits and dietary intake could have contributed to the associations between changes in BMI and academic performance. As such, overweight/obesity is consistently associated with poorer academic performance and weight loss in children with overweight/obesity improves academic performance. Importantly, these relationships may be observed independent of socioeconomic status and lifestyle behaviors, including exercise habits, screen time, and learning duration.

However, whether weight gain in children with underweight is associated with academic performance remains controversial. Nonetheless, in our study, weight gain in children with underweight was also associated with improvement in academic performance. To the best of our knowledge, this study is the first to demonstrate that weight loss in children with overweight/obesity and weight gain in children with underweight are associated with improvement in academic performance in Asian children. Recent longitudinal observational studies that targeted Asian children demonstrated a weak or null relationship between BMI and academic performance^6,24^. In our study, although an interaction effect of baseline BMI and BMI changes on changes in academic performance over 2 years was noted, changes in BMI had no main effect on changes in academic performance over 2 years. These results could partly explain the weak or null association of BMI changes with academic performance in Asian children as previously reported^6,24^. Furthermore, a previous cross-sectional study demonstrated an inverted U-shape relationship between BMI and academic performance in Japanese children^17^. Hence, in Asian children, a nonlinear relationship between BMI and academic performance is possible. Our study extends such finding to a longitudinal relationship where weight gain in children with underweight improves academic performance over 2 years, which is a novel contribution to the literature.

One possible underlying mechanism of the negative relationship of underweight and overweight/obesity to academic performance is associated with worsened executive function^7,8,14–16^, which is closely related to academic performance^12,25^. However, we could not elucidate the underlying mechanisms of the negative relationship of childhood underweight and overweight/obesity to the academic performance from the current findings. Furthermore, the mechanisms behind the associations between underweight and overweight/obesity with altered academic performance and executive function could be different. Hence, further study is warranted to clarify the mechanisms of the negative relationship of childhood underweight and overweight/obesity to academic performance.

The strength of this study is that the relationship between weight change and academic performance was evaluated after controlling for the following confounders that could critically influence academic performance: socioeconomic status, lifestyle behaviors, including exercise habits, screen time, and learning duration, and cardiorespiratory fitness. The positive relationship of favorable change in weight status to the improvement in academic performance was independent of these confounders. Thus, a program that aims to maintain healthy weight in schools and/or community sports club settings may contribute to an enhanced academic performance. An additional strength of our study is the investigation involving children in Japan; previous relevant studies have been mainly conducted in western countries. However, the current findings should be interpreted with caution. Low BMI is considered to reflect both lean and slower maturation. We could not identify the amount of weight gain in lean children or children with delayed growth as an increase in BMI. In addition, the lack of measures/information regarding special education needs, learning disabilities, and/or neurological disorders (e.g., attention-deficit hyperactivity disorder and autism) and puberty status should be acknowledged as limitations of the present study. Future studies are needed to clarify the influence of weight gain on the academic performance of lean children and maturation in children with delayed growth.

In conclusion, weight gain in Japanese children with underweight and weight loss in children with overweight/obesity could improve their academic performance. These relationships were observed after controlling for other critical socioeconomic, physiological, and behavioral factors for academic performance. Importantly, these significant associations were observed not only in underweight and obese children but also in mild underweight and overweight children (≤16.6 and ≥22.3 kg/m^2^). Finally, the positive association of weight optimization to academic performance could be observed regardless of academic subjects and sex. Dissemination of this study’s findings is particularly important for educators and policymakers.

## Methods

### Participants

**Fig. 4:**
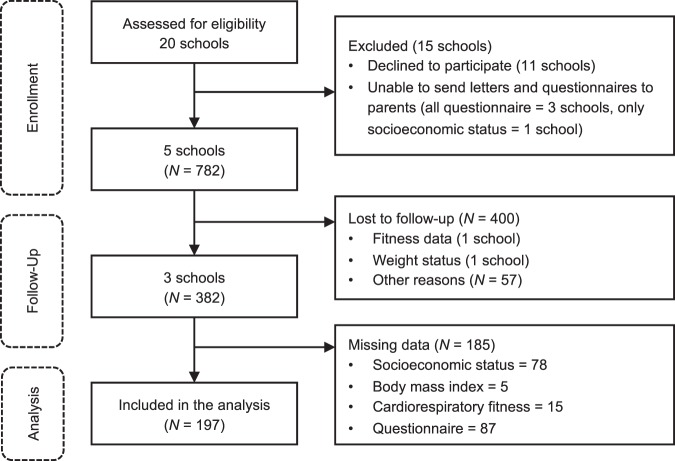
Overview of the recruitment of children and the number of missing data.

Figure 4 shows an overview of the recruitment of children and the number of missing data for each variable. The inclusion criteria for school recruitment were as follows: public junior high schools funded by local governments and schools that had three to six classes per grade. These criteria were set to enroll general junior high school students for a small, biased sample. We asked 20 public junior high schools in Sapporo and cities near Sapporo to participate in this study. Principals of 11 junior high schools declined and of 3 junior high schools did not allow sending of questionnaires asking for data on family environments and socioeconomic status to children’ parents/guardians. Two schools were excluded, because we could not follow-up with them. A total of 197 seventh-grade children completed the 2-year study period (boy = 98, girl = 99). The recruitment procedure has been described in detail elsewhere^17,26^. Briefly, children were given a packet of documents containing a leaflet and questionnaires for themselves, a letter to their parents/guardians explaining the study and requesting their cooperation, and questionnaires for their parents/guardians. The explanation letter informed parents that all data collected from the students and their parents/guardians would be anonymous (i.e., we would not collect personal information such as students’ names and birth dates). Only students whose parents/guardians consented to participation after reading the explanation letter completed and returned the questionnaires. This study was approved by the institutional review board of Hokkaido University of Education and the principals of their children’s schools.

### Academic performance

Academic performance was assessed using total grade points of five school subjects (i.e., Japanese, Mathematics, Social Studies, Sciences, and English). Children earn 1–5 points for each subject; thus, a student can receive a maximum of 25 grade points for the 5 subjects. Grade points of academic subjects in seventh and ninth grades were determined and collected at the end of the school year in March 2013 and March 2015, respectively (the school year in Japan starts in April and ends in March of the following year, i.e., April 2012 to March 2013 and April 2014 to March 2015 are the school years at baseline and follow-up, respectively). A grade point of each academic subject was evaluated by teachers of each academic subject throughout a school year, based on class activities, reflection papers, and paper test scores. Although the contribution rate of each grade point element was not regulated precisely by the national government of Japan, ranges of the contribution rates were provided. Thus, grade-point assessments across schools would be mostly standardized in public junior high schools in this study.

### BMI and cardiorespiratory fitness

BMI was calculated as body weight (kg)/height (m^2^). Cardiorespiratory fitness was assessed using the 20 m shuttle run test^26,27^. The 20 m shuttle run test has been adopted by the Ministry of Education, Culture, Sports, Science, and Technology of Japan and is annually conducted nationwide. Data obtained from the 20 m shuttle run test were converted into a score (maximum of 10) based on Japanese normative data^26^. BMI and physical fitness were evaluated by physical education teachers. BMI was evaluated during May and cardiorespiratory fitness was evaluated during June and July for the seventh- and ninth-grade children.

### Lifestyle behaviors, learning habits for academic school subjects, and socioeconomic status

The children completed a questionnaire concerning their daily lifestyle behaviors, including exercise habits, screen time, and learning duration. Specific details of the questionnaire are shown in the Supplementary Method. Briefly, childrens’ exercise habits were measured according to exercise frequency (days/week, excluding physical education classes), exercise duration on weekdays (excluding physical education classes), exercise duration on weekends, and participation in school-based extracurricular sports clubs. Screen time was defined as the duration of watching TV and using electronic devices (e.g., video games and mobile phones). Learning duration on academic subjects was assessed as the durations of learning after school on weekdays and learning on weekends. For socioeconomic status, participants’ parents completed a questionnaire about household income and maternal educational history. Participants and their parents completed a questionnaire during October for the seventh- and ninth-grade children.

### Statistical analysis

All statistical analyses were conducted with *α* = 0.05 using R Studio software version 1.1.463. Principal components analyses were performed to evaluate questionnaire items of the following components: exercise habits, learning duration, screen time, and socioeconomic status. A positive score indicates greater exercise habits and longer learning duration, less screen time, and high socioeconomic status.

To determine the predictors of change in total grade points of five school subjects over 2 years, a hierarchical multiple regression analysis was performed as a main analysis. The variables were entered into the regression model in the following order: (1) sex (coded as 0 = girls, 1 = boys), socioeconomic status, and total grade points of five school subjects at baseline; (2) exercise habits, learning duration, screen time, BMI, and cardiorespiratory fitness at baseline; (3) changes in exercise habits, learning duration, screen time, BMI, and cardiorespiratory fitness over 2 years; and (4) interaction effects of BMI and changes in exercise habits, learning duration, screen time, BMI, and cardiorespiratory fitness over 2 years. If a significant interaction was found, the Johnson–Neyman Interval was illustrated and a simple slope analysis was performed to examine the main effects of explanatory variables on the total grade points of school subjects when baseline BMI = mean − 1 SD and mean + 1 SD.

To test the moderating effects of sex, we added two- to three-way interaction regarding sex to the regression model in steps 4 and 5, respectively.

To examine the reproducibility and validity of the main analysis, we split the participants into two groups and performed hierarchical multiple regression analysis in each group in the same way as main analyses.

A sensitivity analysis was performed based on the current sample size with 80% power, and *α* of 0.05 demonstrated sufficient sensitivity to detect increased *R*^2^ = 0.04, as computed using G*Power 3.1^28^. Given that a previous study reported increased *R*^2^ = 0.04–0.06 when BMI was entered into the regression model predicting academic performance^7^, we considered that the current sample size is adequate.

### Reporting summary

Further information on research design is available in the Nature Research Reporting Summary.

## Supplementary information

Supplemental material

Reporting summary

## Data Availability

The data that support the findings of this study are available from the corresponding author upon reasonable request.

## Code availability

No custom codes were used in the analysis of this study.
