# Supplementary material for: Longitudinal relationship of favorable weight change to academic performance in children
Source: NPJ Sci Learn. 2020 Apr 24;5:4. doi: 10.1038/s41539-020-0063-z (PMC7181801; doi:10.1038/s41539-020-0063-z)
Supplement: Supplementary file 1 — Supplemental material [file 41539_2020_63_MOESM1_ESM.pdf]

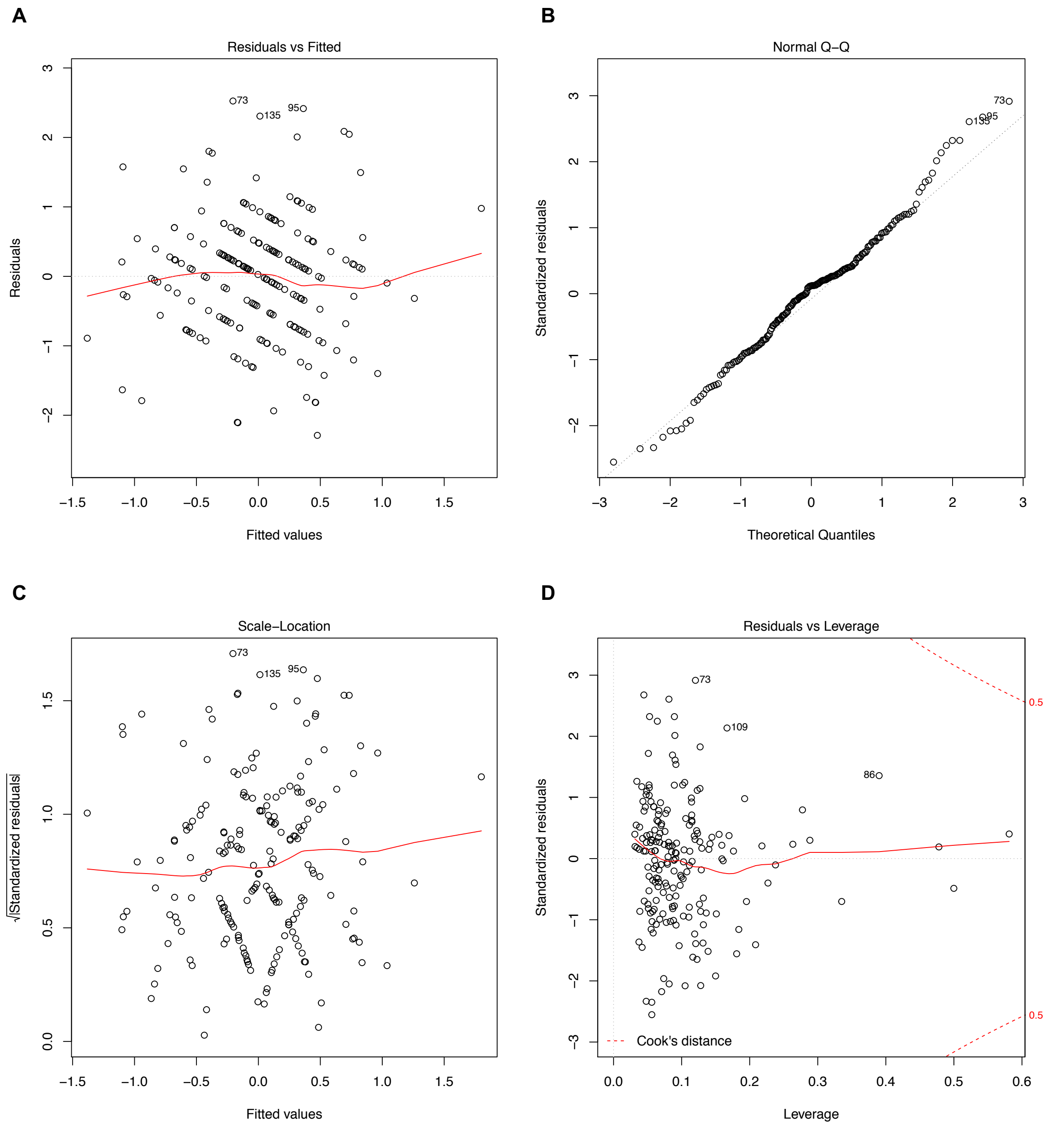

Supplementary Figure 1. Diagnostic plots for multiple linear regression analysis. A. Scatter plot for the regression-predicted values versus the corresponding residuals. The scatter plot for the regression-predicted values versus the corresponding residuals was equally spread around a horizontal line without distinct patterns indicating that there were not non-linear relationships. B. The normal Q-Q diagram. The normal Q-Q plot showed that the residuals follow a straight line, indicating that residuals were normally distributed. C. The scale-location graph. The scale-location plot showed a horizontal line with equally spread points indicating that residuals were spread equally along with the ranges of predictors. D. The Cook's distance diagram. Cook's distance plot showed that there was no outlier that significantly altered current results.

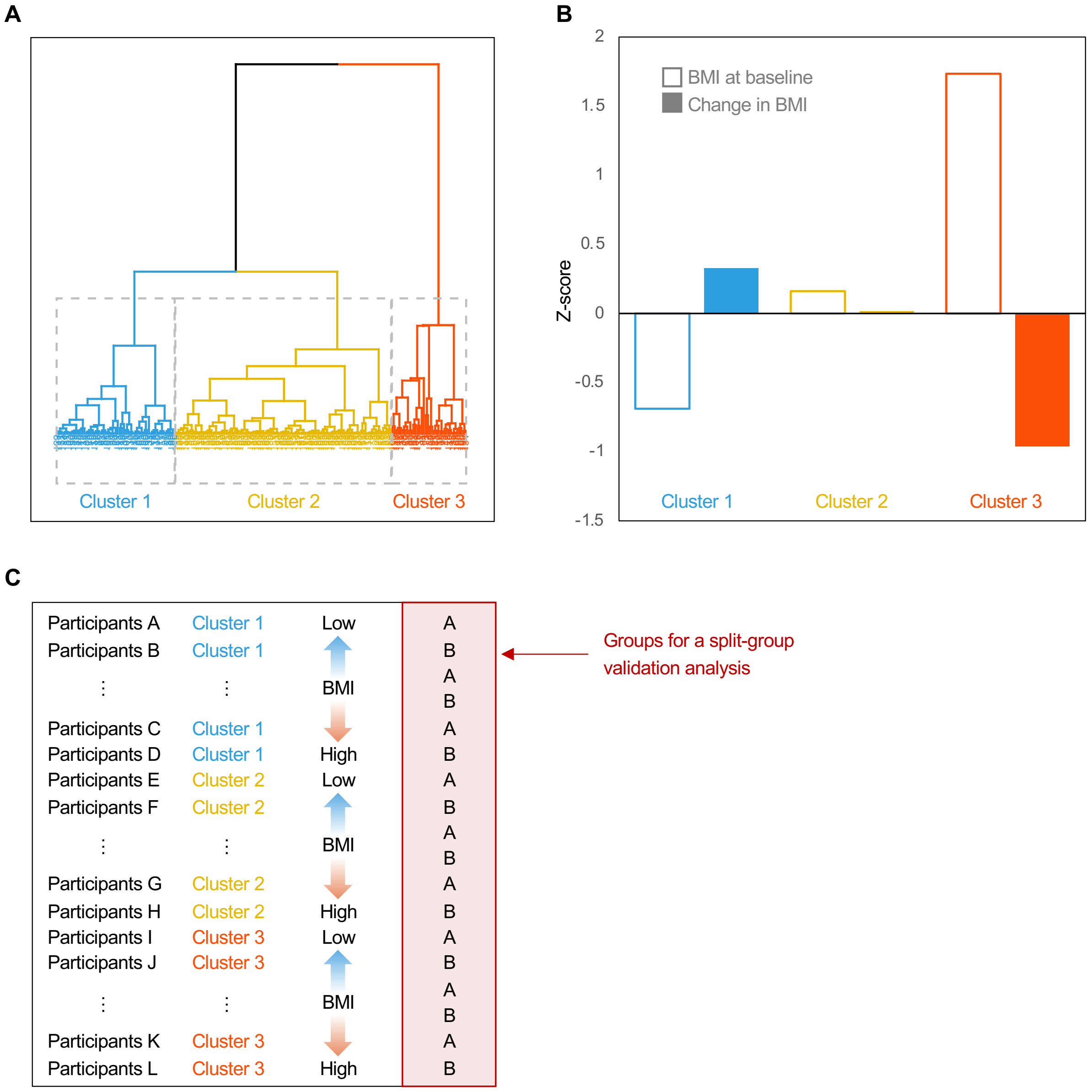

Supplementary Figure 2. Result of the cluster analysis (A and B) and grouping methods for reproducibility and validity analysis.

Supplementary Table. Characteristics of the study participants in each group

	Baseline		2-year follow-up	
	Group A	Group B	Group A	Group B
N (boys / girls)	99 (42/57)	98 (56/42)		
Socioeconomic status	–0.1 ± 1.0	0.1 ± 1.0		
Total grade points of five academic subjects	18.5 ± 3.8	18.4 ± 4.0	18.3 ± 4.2	18.6 ± 4.6
BMI (kg/m^2)	19.1 ± 3.3	19.1 ± 3.2	20.2 ± 2.9	19.9 ± 2.9
Cardiorespiratory fitness	5.1 ± 2.1	4.8 ± 2.0	6.9 ± 2.2	6.7 ± 2.0
Exercise habits (z-score)	0.0 ± 1.0	0.0 ± 1.0	0.0 ± 1.0	0.0 ± 1.0
Screen time (z-score)	0.0 ± 1.0	0.0 ± 1.0	0.0 ± 0.9	0.0 ± 1.1
Learning duration (z-score)	0.1 ± 1.0	–0.1 ± 1.0	0.1 ± 0.9	–0.1 ± 1.1

Values are presented as *N* or mean ± SD. BMI = body mass index.

Supplementary Methohd.

Questionnaires for daily lifestyles, learning habits on academic subjects, and family backgrounds.

How often do you engage in exercise in a usual week, including school-based extracurricular sports clubs (excluding PE class)?

i) 7 days ii) 6 days iii) 5 days iv) 4 days v) 3 days

vi) 2 days vii) 1 day viii) no

For how long do you engage in exercise/sports on a daily basis, school-based extracurricular sports clubs (excluding PE class)?

a. On weekdays

i) Over 4 h ii) 3 to 4 h iii) 2 to 3 h iv) 1 to 2 h

v) 30 min to 1 h vi) 15 to 30 min vii) less than 15 min

b. On weekends

i) Over 4 h ii) 3 to 4 h iii) 2 to 3 h iv) 1 to 2 h

v) 30 min to 1 h vi) 15 to 30 min vii) less than 15 min

In a usual day, how long do you watch TV or videos?

i) Over 2 h ii) 1 to 2 h iii) 30 min to 1 h iv) 15 to 30 min

v) almost no watching

In a usual day, how long do you play video games and/or use your mobile phone?

i) Over 2 h ii) 1 to 2 h iii) 30 min to 1 h

iv) 15 to 30 min v) almost no watching

How long to you spend in learning activities in a usual day (weekday/weekend), including learning in a cram school with a private tutor?

a. On a weekday

i) Over 3 h ii) 2 to 3 h iii) 1 to 2 h iv) 30 min to 1 h v) less than 30 min

b. On weekend

i) Over 3 h ii) 2 to 3 h iii) 1 to 2 h; iv) 30 min to 1 h v) less than 30 min

How much does your family earn annually?

i) Less than ¥2,000,000 ii) ¥2,000,000 to ¥4,000,000 iii) ¥4,000,000 to ¥6,000,000

iv) ¥6,000,000 to ¥8,000,000 v) more than ¥8,000,000

What is the highest level of maternal education?

i) Completed junior high school ii) Completed or dropped out of high school

iii) Completed or dropped out of a vocational school iv) Completed or dropped out of a junior college

v) Completed or dropped out of undergraduate studies vi) Completed or dropped out of graduate studies
